# Intermittent versus continuous esketamine infusions for long-term pain modulation in complex regional pain syndrome: protocol of a randomized controlled non-inferiority study (KetCRPS-2)

**DOI:** 10.1186/s12891-023-06258-4

**Published:** 2023-03-29

**Authors:** Thomas J. P. Mangnus, Maaike Dirckx, Krishna D. Bharwani, Sara J. Baart, Theodora A. M. Siepman, Ken Redekop, Willem A. Dik, Cecile C. de Vos, Frank J. P. M. Huygen

**Affiliations:** 1 Department of Anesthesiology, Center for Pain Medicine, Erasmus MC University Medical Center, Rotterdam, the Netherlands; 2 Department of Biostatistics, Erasmus MC University Medical Center, Rotterdam, the Netherlands; 3 Institute of Health Policy & Management, Erasmus University, Rotterdam, the Netherlands; 4 Laboratory Medical Immunology, Department of Immunology, Erasmus MC University Medical Center, Rotterdam, the Netherlands

**Keywords:** CRPS, Complex regional pain syndrome, Inpatient, Outpatient, Esketamine, Ketamine, KetCRPS-2, Study protocol

## Abstract

**Background:**

Complex regional pain syndrome (CRPS) is a chronic pain condition of an extremity. While achieving pain relief in CRPS is challenging, esketamine infusions can accomplish pain relief for several weeks post-infusion in a subgroup of CRPS patients. Unfortunately, CRPS esketamine protocols are very heterogeneous in advice on dosage, administration and treatment setting. Currently, no trials are available that study differences between intermittent and continuous esketamine infusions for CRPS. With the current situation of bed shortages, it is difficult to admit patients for several consecutive days for inpatient esketamine treatments. In this study, we investigate whether 6 intermittent outpatient esketamine treatments are not inferior to a continuous 6-day inpatient esketamine treatment in establishing pain relief. In addition, several secondary study parameters will be assessed in order to investigate mechanisms responsible for pain relief by esketamine infusions. Furthermore, the cost-effectiveness will be analyzed.

**Methods:**

In this RCT, the primary objective is to demonstrate that an intermittent esketamine dosing regimen is non-inferior to a continuous esketamine dosing regimen at 3 months follow-up. We will include 60 adult CRPS patients. The inpatient treatment group receives a continuous intravenous esketamine infusion for 6 consecutive days. The outpatient treatment group receives a 6-hour intravenous esketamine infusion every 2 weeks for 3 months. Esketamine dose will be individually tailored and is started at 0.05 mg/kg/h and can be increased to a maximum of 0.2 mg/kg/h. Each patient will be followed for 6 months. The primary study parameter is perceived pain intensity, measured by an 11-point Numerical Rating Scale. Secondary study parameters are conditioned pain modulation, quantitative sensory testing, adverse events, thermography, blood inflammatory parameter, questionnaires about functionality, quality of life and mood and costs per patient.

**Discussion:**

If our study reveals non-inferiority between intermittent and continuous esketamine infusions, these findings can be beneficial to increase the availability and flexibility of esketamine infusions through outpatient treatments. Furthermore, the costs of outpatient esketamine infusions could be lower than inpatient esketamine infusions. In addition, secondary parameters may predict response to esketamine treatment.

**Trial registration:**

ClinicalTrials.gov Identifier NCT05212571, date of registration 01-28-2022.

Protocol version: Version 3, February 2022.

**Supplementary Information:**

The online version contains supplementary material available at 10.1186/s12891-023-06258-4.

## Background

Complex regional pain syndrome (CRPS) is described by the International Association for the Study of Pain (IASP) as “a syndrome characterized by a continuing regional pain that is seemingly disproportionate in time or degree to the usual course of pain after trauma or other lesion. The pain is regional and usually has a distal predominance of abnormal sensory, motor, sudomotor, vasomotor, edema and/or trophic findings” [1]. CRPS can have a severe impact on quality of life of patients and can lead to substantial physical as well as social disability [2]. When treating CRPS, the most prominent pathophysiological mechanisms of CRPS should be targeted in an individually tailored manner [3]. Several underlying pathophysiological mechanisms of CRPS have been implicated such as inflammation, peripheral and central sensitization, vasomotor disturbances and motor disturbances [3].

A pharmacotherapeutic option for targeting the pathophysiological mechanism central sensitization in CRPS is the dissociative anesthetic ketamine [3]. Ketamine is a racemic mixture of the enantiomers R-ketamine and S-ketamine (esketamine). Esketamine is preferred in the management of CRPS as esketamine possesses respectively twofold and fourfold stronger anesthetic and analgesic properties than R-ketamine and racemic ketamine [4, 5]. Evidence suggests a part of the analgesic effect of esketamine is mediated by antagonizing N-methyl-D-aspartate (NMDA) receptor activation and thereby influencing sensory processing disturbances [3, 6]. Some studies have also attributed immunoregulatory effects to esketamine [7–9]. Therefore, esketamine may inhibit the inflammatory pathophysiological mechanism of CRPS.

The effects of esketamine on pain relief in CRPS patients have been reviewed by Zhao et al. [10]. The investigators concluded that intravenous esketamine therapy for CRPS can provide effective pain reduction for up to 3 months post infusion in a subgroup of CRPS patients [10]. Unfortunately, CRPS literature contains a wide range of esketamine dosing regimens with the result that clinical protocols on dosage and administration are very heterogeneous [5, 10, 11].

In the Netherlands, CRPS patients are treated according to the Dutch guideline for CRPS [12]. This guideline recommends esketamine treatment when the pain is severe (Numerical Rating Scale ≥7) and other more conservative therapies have failed. Both outpatient and inpatient esketamine treatments are offered in the Netherlands [5]. The clinical protocol of our Center for Pain Medicine is a 6-day inpatient admission for continuous esketamine administration, with almost 50% of the patients still reporting pain relief 4 weeks after treatment [13]. Unfortunately, bed shortages and financial boundaries limit inpatient esketamine infusions. In addition, the COVID-19 pandemic showed that hospital bed capacity is limited and esketamine infusions for CRPS have been postponed or cancelled due to the scarcity of hospital beds and health care professionals during the pandemic. Esketamine infusions in an outpatient setting might increase flexibility and availability of esketamine treatment and may reduce costs. However, differences between intermittent and continuous esketamine infusions for CRPS have never been compared in randomized controlled trials (RCTs). To study this, we will conduct a non-inferiority RCT to assess whether a series of intermittent esketamine infusions, in 3 months every 2 weeks a day-care outpatient infusion of 6 hours, is non-inferior to a 6-day inpatient admission with continuous esketamine administration in establishing pain relief in CRPS patients. In addition, in both treatment settings, several objective parameters will be assessed to investigate mechanisms responsible for pain relief. Hopefully, this will lead to the identification of predictors of response to esketamine and in the future CRPS patients will be selected for esketamine treatment in a mechanism-based manner. Furthermore, the results of this study will include a cost-effective analysis. This study will help to guide decisions about the use of esketamine treatment for CRPS patients.

## Methods

### Ethics

Ethical approval for this study was obtained from the local Medical Ethics Committee (MEC-2021-0426). The EudraCT number is 2021-000640-21. The trial is registered in the Clincialtrials.gov registry (NCT05212571). Important protocol modifications will be reported to the local Medical Ethics Committee. Eligible patients can participate in the study after informed consent is obtained. Additional consent is requested for collection and use of participant data and biological specimens in future studies in the informed consent form. All data will be handled confidentially according to the General Data Protection Regulation.

### Study setting

The Center for Pain Medicine is a tertiary referral pain center specialized in the diagnosis and treatment of CRPS. The setting of the study will be in the outpatient clinic of the Center for Pain Medicine or the inpatient ward at the Center for Pain Medicine.

### Eligibility criteria

In order to be eligible to participate in this study, patients must meet the new IASP diagnostic criteria for CRPS [14] or must have met the new IASP diagnostic criteria of CRPS in the past (‘CRPS with Remission of Some features’) [15]. Inclusion criteria are age ≥ 18 years, CRPS in one upper extremity and/or CRPS in one lower extremity, esketamine treatment in an elective setting (i.e. not in an emergency setting), adequate comprehension of the Dutch language and willingness and ability to participate in the study. Patients are selected for an intravenous esketamine treatment by their treating pain specialist according to the Dutch CRPS guidelines [12]. Patients must suffer severe pain and/or therapy-refractory CRPS (i.e. when other more conservative treatments have failed).

Contraindications to participate in this study are based on the United States (US) consensus guidelines on the use of intravenous ketamine infusions for chronic pain [16]: severe liver disease, schizophrenia, psychosis, delirium, manic depression, active substance abuse, intoxication with alcohol or other substances, poorly controlled hypertension, unstable angina, high-risk coronary vascular disease, heart failure, elevated intracranial pressure, elevated intraocular pressure, thyrotoxicosis, pregnancy. Furthermore, use of derivatives of xanthine (such as theophylline) or ergometrine are contraindicated because of interactions with esketamine. For each patient, the contraindications and precautions for use of esketamine infusions will be assessed by their treating pain specialist.

### Recruitment, allocation and blinding

CRPS patients who are selected for a treatment with intravenous esketamine will be placed on the regular clinical waiting list of the Center for Pain Medicine. Patients will be selected chronologically from this waiting list for esketamine treatment and are contacted by a researcher for trial information. The expected duration of the study inclusion period will be approximately 3 years. The first patients will be included in 2022. Patients can withdraw from the study at any time without giving reasons. This is without any consequences for further medical treatment. Patients who are not willing to participate in this study will receive standard care (inpatient esketamine infusions).

After informed consent, patients will be enrolled and randomized using Castor software (Castor EDC). Previous treatments with esketamine may influence the outcomes of this clinical trial and therefore stratified randomization is performed with randomization blocks according to whether patients received previous esketamine treatment. The randomization will be done with an allocation ratio 1:1. The patients, researchers and statistician will not be blinded during the data collection and analysis. The researcher will enroll patients and will assign patients to the interventions after randomization.

### Participant timeline

**Fig. 1:**
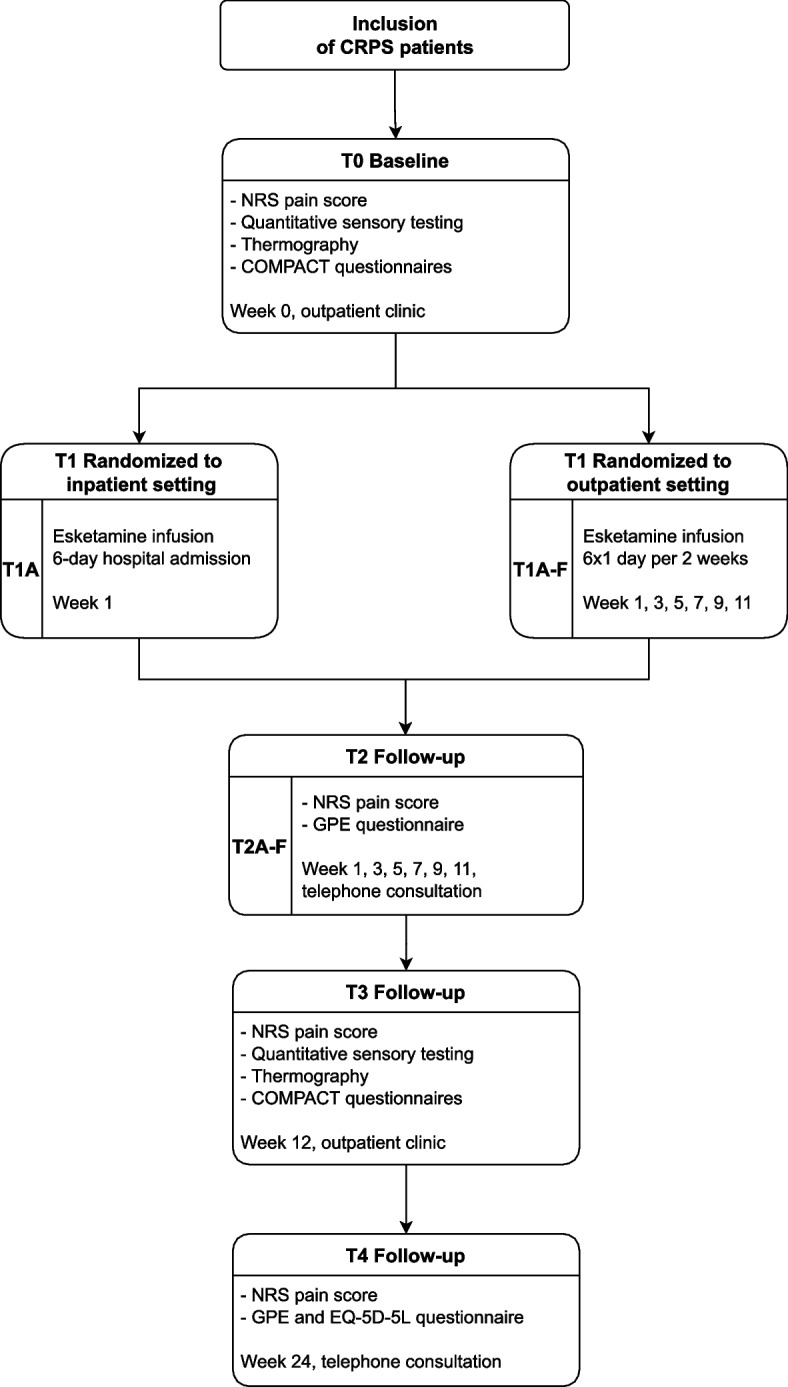
Detailed flowchart of the KetCRPS-2 study *Abbreviations: Complex Regional Pain Syndrome (CRPS), Numerical Rating Scale (NRS), Global Perceived Effect* *(GPE), the Core Outcome Measurement set for complex regional PAin syndrome Clinical sTudies (COMPACT*)

During this study, endpoints will be assessed at baseline (T0), during inpatient and outpatient esketamine treatments (T1), the day after each hospital admission (T2) and at 3 months (T3) and 6 months (T4) after the first esketamine infusion. The intermittent 6 outpatient esketamine treatments are addressed with T1A, T1B, T1C, T1D, T1E and T1F. The continuous inpatient treatment is addressed with T1A. Figure 1 provides the flowchart of the study. Furthermore, Table 1 presents an overview of schedule of enrolment, interventions and assessments according to standard protocol items recommendations for interventional trials (SPIRIT) during the entire course of this study [17, 18]. All visits and interventions will be discussed in detail below.

**Table 1:** Overview of schedule of enrolment, interventions and assessments in the KetCRPS-2 study. *According to Standard Protocol Items Recommendations for International Trials (SPIRIT)* [17, 18]

TIMEPOINT	STUDY PERIOD						
	Enrolment	Allocation	Post-allocation				Close-out
	***-t***_***2***_	***-t***_***1***_	***T0***	***T1A-F***	***T2A-F***	***T3***	***T4***
**ENROLMENT**							
**Eligibility screen**	X						
**Informed consent**	X						
**Allocation**		X					
**INTERVENTIONS**							
***Esketamine infusion***				X			
**ASSESSMENTS**							
***Medical history***	X		X			X	X
***NRS pain scores***			X	X	X	X	X
***CRPS severity score*** [14]			X			X	
***COMPACT questionnaire(s)*** [19]			X		X^a^	X^c^	X^a^
***Quantitative sensory testing***			X			X	
***Conditioned pain stimulation***			X			X	
***Thermography***			X			X	
***Blood samples***			X	X^b^		X	

*Abbreviations*: *NRS* Numerical Rating Scale, *GPE* Global Perceived Effect, *COMPACT* the Core Outcome Measurement set for complex regional PAin syndrome Clinical sTudies

^a^Only the Global Perceived Effect [20] and/or the EQ-5D-5L [21]

^b^Liver enzymes are assessed to detect hepatotoxicity (standard care)

^c^QST features temporal summation and pressure algometry

### Interventions

The esketamine treatment protocol differs for the continuous inpatient and intermittent outpatient treatment arms. The inpatient protocol is derived from a study by Sigtermans et al. [22]. For the inpatient treatment arm, esketamine is administered intravenously in CRPS patients for 6 consecutive days. Esketamine dose will be started at 0.05 mg/kg/h and can be increased to a maximum of 0.2 mg/kg/h. The dose can be increased with steps of 0.05 mg/kg/h every hour. For the outpatient treatment arm, esketamine will be administered for 6 hours. Esketamine dose will be started at 0.05 mg/kg/h and can be increased to a maximum of 0.2 mg/kg/h. The dose can be increased with steps of 0.05 mg/kg/h every hour. The increase of the esketamine dose depends on whether the patient experiences pain reduction or side effects. Once the patient notices a reduction in pain, the esketamine dose is not increased further for the duration of their admission. The investigators consider this the effective dose. If the effective dose in an earlier treatment session was higher than 0.05 mg/kg/h, the effective dose can be used as the starting dose in the following outpatient treatments.

If the patient experiences debilitating side effects the dose is reduced until the side effects disappear. Psychomimetic effects of esketamine will be treated with intravenous benzodiazepines or clonidine if necessary. In case of nausea and/or vomiting, intravenous granisetron will be administered. If the side effects subside, the esketamine dose is increased again and effects and side effects are closely monitored. If debilitating side effects persist, the esketamine therapy is discontinued. Side effects, vital parameters and pain scores will be frequently monitored by a nurse. As esketamine can induce hepatotoxicity, liver enzymes are monitored before administration and at the third day of the inpatient treatment or at the third outpatient esketamine treatment (aspartate aminotransferase (AST), alanine aminotransferase (ALT), lactate dehydrogenase (LDH), alkaline phosphatase (AF) and gamma-glutamyl transferase (GGT) [23].

### Outcomes

The primary outcome parameter is perceived pain intensity measured by Numerical Rating Scale (NRS) at T3 and will be compared to the baseline NRS pain scores (T0). The NRS ranges from 0 (no pain) to 10 (worst pain imaginable). Both the current NRS pain score and the average NRS pain score of the last 24 hours will be asked during all study visits.

Several secondary outcome parameters will be assessed. First, sensory-discriminative dimensions of pain are assessed by using quantitative sensory testing (QST) (T0, T3) [24]. QST is a standard set of tests to characterize how somatosensory stimuli are processed and is also used to examine which types of nerve fibers might be damaged [24]. Second, information on the endogenous pain inhibitory pathway of CRPS patients will be assessed with conditioned pain modulation (T0, T3) [25]. Third, inflammatory activity will be assessed by measuring the serum level of soluble IL-2 receptor (sIL-2R) (T0, T3). sIL-2R is a surrogate marker for T-cell activation and may be used as a biomarker for monitoring T-cell involvement in CRPS-related inflammation [26, 27]. Fourth, temperature asymmetry will be measured by thermographic imaging at T0 and T3. Thermographic imaging was previously shown to be a reliable additive diagnostic tool to determine temperature differences between the affected and contralateral extremity in CRPS patients [28]. Fifth, pain intensity, syndrome severity, participation and function, emotional and physiological function, self-efficacy, catastrophizing and patient global perceived effect are assessed using the questionnaires of the core outcome measurement set for complex regional pain syndrome clinical studies (COMPACT) (T0, T2, T3, T4) [19]. The COMPACT questionnaires are defined by an international consortium of patients, clinicians and researchers as minimum core set of standardized, patient-reported questionnaires that cover important domains about the presentation and course of CRPS and factors that influence the syndrome (see Table 2) [19]. Sixth, the (effective) esketamine dose during infusion (T1). Seventh, adverse events related to esketamine treatment: dysphoria, euphoria, hallucinations, nightmares and vivid dreams, anxiety, agitation, blurry vision or diplopia, nausea or vomiting, sedation, hepatic toxicity, headache, phlebitis, infiltration/extravasation and dislodgement of peripheral intravenous catheter (T1, T2). Eight, number of administered co-interventions (benzodiazepines, clonidine, granisetron) related to adverse events (T1). Ninth, changes in the dose of pain medication during study enrolment (T0, T3, T4). Tenth, resource use and costs per patient will be assessed. Eleventh, changes in participation in employment, education or voluntary work during study enrolment (T0, T3, T4). Last, the difference in NRS pain scores over time at T1, T2, T3 and T4. The results of baseline tests or parameters will serve as a baseline and all subsequent evaluations will be compared with these results.

**Table 2:** Questionnaires used in KetCRPS-2 study. *Adapted from the Core Outcome Measurement set for complex regional PAin syndrome Clinical sTudies (COMPACT)* [19]

COMPACT questionnaires [19]	Details	Study period assessed
Global Perceived Effect (GPE) [20]	The Global Perceived Effect asks the patient to rate, on a numerical scale, how much their condition has improved or deteriorated since some predefined time point.	T2, T3, T4
PROMIS-29 Profile version 2.1 [29]	Assesses 7 domains, each with 4 questions: depression, anxiety, physical function, pain interference, fatigue, sleep disturbance, and ability to participate in social roles and activities.	T0, T3
Short-form McGill Pain Questionnaire-2 (SF-MPQ-2) [30]	Six neuropathic items capturing the quality of pain	T0, T3
Pain Catastrophizing Scale [31]	To measure how catastrophizing affects the pain experience.	T0, T3
EQ-5D-5L [21]	To measure health state, comprising mobility, self-care, usual activities, pain/discomfort, anxiety/depression	T0, T3, T4
Pain Self-Efficacy Questionnaire [32]	The respondent considers how confident they are performing each activity, while taking their pain into account	T0, T3
CRPS severity score [14]	CRPS symptoms and signs based on the Budapest diagnostic clinical criteria	T0, T3

### Baseline measurement T0

The patients are invited to the Center for Pain Medicine for a baseline measurement. Information on demographics, CRPS-affected limb(s), CRPS duration and medication is obtained during history taking. The patients will be asked for NRS pain scores. During the baseline visit, the CRPS severity score will be assessed [14]. At both T0 and T3, peripheral blood samples will be taken to obtain information on the serum sIL-2R level and the total set of the COMPACT questionnaires will be completed [19].

QST at the Center for Pain Medicine is measured according to the protocol by Rolke et al. and provides diagnostic information on the somatosensory profile of the CRPS affected area [24]. At T0, the entire QST protocol by Rolke et al. is conducted to give information on the baseline somatosensory profile of the included patients. In addition, the protocol by Rolke et al. [24] may be used in CRPS as a diagnostic test for phenotyping different CRPS subgroups. QST consists of thermal, pressure, mechanical sensation and pain thresholds and these stimuli are measured using thermodes, pinpricks, Von Frey filaments, electrical stimulation and an algometer. All tests will first be performed on the clinically unaffected contralateral side before testing on the CRPS-affected side. The punctum maximum of pain on the CRPS-affected side was used as the reference for the anatomical testing location and will also be used on the clinically unaffected contralateral side.

The conditioned pain modulation protocol used in this RCT is based on the protocol used at the pain department of Radboud University Nijmegen, the Netherlands [33, 34] and adapted from Kriek et al. [35] at our department. All conditioned pain modulation tests are done in a standardized order. We use electrical stimulation and pressure algometry for the test stimuli. For electrical stimulation, the current perception threshold (CPT) and pain perception threshold (PPT) are determined three times with an ascending electrical stimulation protocol at the upper extremity at the contralateral side of the affected CRPS extremity. The conditioned pain modulation protocol is enriched with an additional test stimulus according to Yarnitsky et al. [36] by using pressure algometry. For pressure algometry, the current perception threshold (CPT) and pain perception threshold (PPT) are determined three times with an ascending pressure algometry protocol at the trapezius muscle. The ipsilateral healthy extremity of the CRPS affected extremity is submerged in ice water as the conditioning stimulus. For the full conditioned pain modulation protocol see Additional file 1. Our conditioned pain modulation analysis method is adapted from Olesen et al. [37] and Kriek et al. [35]. The PPTs will be determined three times, and the median value of these three values will be calculated for further analysis. By taking medians, outliers will not be taken into analysis. Ratios will be used to investigate the relationship of the threshold between the affected CRPS side and the healthy contralateral side [35, 37]. The advantage of using ratios is that they eliminate the intersubject differences in absolute CPT and PPT [35]. The ratio of each PPT threshold is calculated by using the formula (threshold CRPS affected side) / (threshold healthy contralateral side) [35]. This yields the CPT ratio and PPT ratio of the electrical stimulation and pressure algometry.

At the Center for Pain Medicine, a thermal imaging camera is used in standard clinical practice to measure skin temperature and detect temperature asymmetry in CRPS patients. In this study, thermography will be conducted to detect temperature differences that may be caused by esketamine treatment. Specialized researchers use a camera from FLIR Advanced Thermal Solutions to detect temperature asymmetry (FLIR T1020).

### Esketamine treatment (T1A-F)

The inpatient treatment arm receives continuous intravenous esketamine infusions for 6 consecutive days during a hospital admission (T1A). The outpatient arm will receive 6 esketamine infusions: one 6-hour infusion every 2 weeks for 3 months, in a day-care setting (T1A-F). Esketamine will be administered by a trained nurse under supervision of a pain specialist.

### Follow-up measurement T2A-F

All patients will be called every 2 weeks (T2A-F). They will be asked for their NRS pain score and if they have experienced any side effects of the esketamine treatment. In addition, the questions of the Global Perceived Effect will be asked [20].

### Follow-up measurement T3

At the 3-months follow-up measurement (T3), the same set of measurements conducted at baseline will be repeated. Only for the QST, a limited protocol will be performed to decrease patient burden: only temporal summation, pressure algometry and conditioned pain modulation will be assessed to provide information on pain processing. Our research group prefers to conduct only specific subsets that are suggested to be effected by esketamine treatments and give information on pain processing. The features temporal summation, pressure algometry and conditioned pain modulation might predict treatment outcome of pain relief by esketamine treatment [38–40]. Bosma et al. showed that neuropathic pain patients who have enhanced temporal summation of pain benefit from treatment with ketamine [38]. With regard to pressure algometry, Kirkpatrick et al. reported that clinical outcomes correlated with improvement in pain thresholds by pressure algometry in CRPS patients [40]. They suggested that pain thresholds by pressure algometry are a valid method to measure pain in CRPS as well as to measure clinical outcome after treatment with ketamine [40]. Furthermore, Niesters et al. reported enhanced conditioned pain modulation responses in neuropathic pain after ketamine infusion [39]. The observed treatment effects in neuropathic pain patients suggest a role for conditioned pain modulation engagement in analgesic efficacy of ketamine treatment [39]*.*

### Follow-up measurement T4

At 6-months, patients will be called for their last follow-up measurement (T4). They will be asked for their NRS pain score, the Global Perceived Effect [20] and the EQ-5D-5L [21]. After the end of the study, all participants will continue to receive standard follow up medical care for their CRPS.

### Sample size

Three months after the inpatient treatment the investigators expect a reduction of the average NRS pain score for pain from 8.0/10 to 6.0/10 (SD 1.5) and for the outpatient group from 8.0/10 to 6.0/10 (SD 1.5). These expectations are derived from a retrospective study of the inpatient continuous esketamine treatment for CRPS patients at the Center for Pain Medicine [13]. If there is truly no difference between the continuous esketamine treatment and the intermittent outpatient esketamine treatment, then 56 patients are required to be 80% sure that the lower limit of a 95% confidence interval will be above the non-inferiority limit of − 1.0. We aim to include 60 CRPS patients that will be randomized between outpatient and inpatient esketamine treatment.

### Statistical methods

Descriptive statistics are used to determine the frequency distributions of demographic variables, and to describe measures of central tendency and of dispersion, dependent on the shape of their distribution. The Shapiro-Wilk test is used to analyze whether or not continuous parameters are normally distributed. The effects of the treatment on the different study parameters will be evaluated with regression models to account for the stratification factor, received esketamine treatment in the past, in the randomization process. Continuous outcomes will be analyzed with a linear regression model, where the residuals will be evaluated for normality. In case of a violation of the assumption, (logarithmic) transformations will be applied. Count outcomes will be analyzed with Poisson regression models and binary outcomes with logistic regression models. For repeated measurements mixed effects, models will be used to account for correlations from measurements within the same patient. No correction of the rejection zone will be made for multiple comparisons, because of the more explorative nature of the analyses of the secondary and other parameters. All analyses will be conducted in accordance with the intention-to-treat principle. Data will be analyzed using the latest version of SPSS and R.

### Data collection methods and management

The study researchers have followed Castor training workshops and have followed the Basic Course Regulation and Organization for Clinical Investigators. The study researchers will obtain informed consent from potential trial participants. An identification code is assigned to the patient. The key to the code is safeguarded by the researcher. All data will be recorded in Case Report Forms (CRF) in Castor. If a participant terminates the trial early, the reason for dropping out, if provided, will be documented. Only the researchers will have access to the final trial dataset.

### Economic evaluation

An economic evaluation will be performed with a health care perspective according to the Dutch national guidelines for economic evaluation [41]. The time horizon of the economic evaluation will correspond to the trial follow-up of 6 months. A cost-effectiveness and a cost-utility analysis will be conducted. Incremental cost-effectiveness ratios will be calculated by dividing the difference in mean costs by the difference in mean effects of the intermittent and continuous esketamine treatment groups. For the effects, the parameters pain intensity measured by the NRS pain score and the Global Perceived Effect [20] will be used. Cost-utility will be based on the EQ-5D-5L [21] and expressed in costs per quality-adjusted life years (QALYs). Bootstrapping techniques will be used to estimate statistical uncertainty and the multiple imputation by chained equation approach will be used for missing data in the economic evaluation.

Healthcare utilization includes hospitalizations, outpatient visits, procedures, diagnostic tests and the prescription of pain medication. These data will be gathered during the prospective study. The costs of healthcare utilization will be valued using Dutch standard costs and prices provided by professional organizations such as the National Health Care Institute if standard costs are not available. The costs of medication will be valued using the prices of the Royal Dutch Society of Pharmacy.

### Monitoring

Monitoring of the data will be performed by an independent monitor. A data safety monitoring board is not needed since this study poses a negligible risk.

Adverse events will be assessed during study visits by the researchers, including the probability that adverse events are associated with the treatment in this study. All adverse events reported spontaneously by the patient or observed by the researchers or clinicians will be recorded. Serious adverse events (SAEs) will be reported within 15 days after they are reported to the researchers. SAEs will be followed until they resolve or reach a stable non-serious level. Depending on the event, follow up may require additional tests or referral to the general physician or a medical specialist.

## Discussion

There is evidence that esketamine infusions can provide clinically effective pain relief in a subgroup of CRPS patients with refractory pain [10, 11]. To our best knowledge, intermittent and continuous esketamine treatments have never been compared in RCTs. This article describes the rationale and design of an RCT that assesses the differences between intermittent and continuous intravenous esketamine treatments in CRPS patients. This study will provide information on whether 6 intermittent outpatient esketamine infusions are non-inferior to a 6-day inpatient continuous esketamine infusion.

As an alternative to lengthy inpatient hospital admissions, an outpatient treatment regimen with multiple clinic visits could be proposed [16, 42–45]. If our study reveals non-inferiority of intermittent versus continuous esketamine infusions, these findings can be beneficial to increase the availability of treatment with esketamine infusions. In addition, patient burden can be reduced by offering esketamine in a more flexible day-care treatment compared to a 6-day consecutive hospital admission. Economic evaluation may show that intermittent outpatient esketamine treatments are more cost-effective.

In this RCT, the intermittent outpatient treatment protocol was defined as 6 day-care esketamine infusions every 2 weeks for 3 months. Our research group decided to incorporate 6 outpatient day-care infusions in our outpatient treatment protocol because our inpatient treatment protocol also consists of 6 days. In addition, we decided to spread the 6 outpatient infusions over 3 months because pain relief is expected to last up to 3 months post-infusion [10, 22]. Furthermore, the intermittent 6 outpatient ketamine infusions over the course of 3 months might be a way to extend the pain relief as result of esketamine administration.

Regarding the intermittent infusion duration, our research group decided to administer esketamine for 6 hours. This is the longest infusion time possible in our outpatient clinic. Unfortunately, there is no gold standard for the duration of esketamine administration. Maher et al. noted that although the general trend is that longer durations of esketamine administration provide increased duration of pain reduction, there may be an optimal infusion duration of several hours beyond which no benefit is derived but the potential for side effects increases [42]. In addition, Noppers et al. described that infusion durations shorter than 2 hours were unlikely to accomplish pain relief for more than 2 days [46]. Furthermore, in 2 survey studies on esketamine in CRPS, the most often reported infusion duration was 4 hours [47] and 6 hours [5]. This is in line with the outpatient treatment protocol of the Center for Pain Medicine.

Regarding the esketamine dose, there is significant heterogeneity between published studies on CRPS [10, 11]. Most of the studies reported esketamine dose based on body weight, with doses ranging from 0.15 to 7.0 mg/kg/hour [11]. In both the inpatient and outpatient treatment protocol of the Center for Pain Medicine, the esketamine dose can be titrated from 0.05 to 0.2 mg/kg/hour. Although, this dosage scheme is relatively low compared to other studies [10, 11], we believe that high dosing of esketamine is not necessary to achieve sufficient pain relief. First, a retrospective study by our research group showed that the median effective esketamine dose in our population was 6 mg/h, which corresponds to 0.1 mg/kg/hour for a 60 kg patient [13]. Furthermore, it is suggested that the effective esketamine treatment for CRPS is not related to the actual dose or rate of the esketamine infusion, as low-dose esketamine regimes showed comparable duration of pain relief compared to high-dose esketamine regimens performed in intensive care units [42].

To predict the response to esketamine infusions more knowledge is required on mechanisms resulting in pain reduction by esketamine infusions [3, 13]. Multiple mechanisms of actions have been proposed for esketamine treatments [3, 48]. Interestingly, upon the termination of esketamine infusions, Sigtermans et al. showed that concentrations of esketamine and its metabolite norketamine rapidly declined [22]. Therefore, it is suggested that the long-lasting analgesic effect of esketamine in CRPS is not driven by pharmacokinetics of esketamine or norketamine [22, 49]. It is likely that long-lasting pain relief is the result of neurotrophic effects at spinal and supraspinal sites that effectively counteract central sensitization by prolonged NMDA receptor desensitization [22, 49]. In addition, the immunoregulatory effects of esketamine may interact with the affected and closely linked immune and nervous system in CPRS patients [7, 50]. For instance, recent studies on depression and comorbid pain described the immunoregulatory aspects of esketamine that may result in downregulation of peripheral pro-inflammatory cytokines and the attenuation of central glial cells [8, 51, 52].

The strengths of this study design are the randomized design and the stratification of patients based on whether they have had esketamine infusions in the past. Another strength of this study is that several objective parameters for esketamine treatments and the cost-effectiveness are assessed. Complementary to further understandings of esketamine mechanisms, diagnostic tests and biomarkers are needed to predict response on esketamine infusions for CRPS patients [13, 27, 53]. By incorporating a peripheral blood inflammatory parameter, conditioned pain modulation, thermography and QST, our research group hopes to provide information on differences between responders and non-responders to esketamine treatment. This paves the way to a more mechanism-based approach in selecting CRPS patient for esketamine treatment. A potential pitfall of the study protocol is that, although CRPS is a multi-mechanism syndrome, patients receive esketamine as a monotherapy. Targeting only a single mechanism might not be sufficient in CRPS [3]. Furthermore, patients cannot be blinded for an intermittent or continuous esketamine treatment. Of note, although the decisions of our research group on the treatment protocols for esketamine administration have been made after thorough consideration, significant uncertainty exists for the most ideal treatment regimen for esketamine infusions and may also differ for each individual CRPS patient. We acknowledge that all decisions on the length and dose of esketamine administration can influence the outcome parameters in this RCT.

## Supplementary Information

**Additional file 1:** Explanation of the full conditioned pain modulation protocol.

## Data Availability

Not applicable.

## Abbreviations

AF: Alkaline phosphatase
ALT: Alanine aminotransferase
AST: Aspartate aminotransferase
COMPACT: Core Outcome Measurement set for complex regional PAin syndrome Clinical sTudies
CRF: Case Report Forms
CRPS: Complex Regional Pain Syndrome
GGT: Gamma-glutamyl transferase
IASP: International Association for the Study of Pain
LDH: Lactate dehydrogenase
MC: Medical Center
NMDA: N-methyl-D-aspartate
NRS: Numerical Rating Scale
QST: Quantitative Sensory Testing
RCT: Randomized Controlled Trial
SPIRIT: Standard Protocol Items Recommendations for Interventional Trials

## References

[1] Classification of chronic pain, Second Edition (revised) IASP Task Force on Taxonomy. https://www.iasp-pain.org/publications/free-ebooks/classification-of-chronic-pain-second-edition-revised/. Accessed 1 Mar 2022.

[2] de Mos M, Sturkenboom MC, Huygen FJ (2009). Current understandings on complex regional pain syndrome. Pain Pract.

[3] Mangnus TJP, Bharwani KD, Dirckx M, Huygen FJPM. From a symptom-based to a mechanism-based Pharmacotherapeutic treatment in complex regional pain syndrome. Drugs. 2022. 10.1007/s40265-022-01685-4 PMC9016036 35247200

[4] Peltoniemi MA, Hagelberg NM, Olkkola KT, Saari TI (2016). Ketamine: a review of clinical pharmacokinetics and pharmacodynamics in anesthesia and pain therapy. Clin Pharmacokinet.

[5] Mangnus TJP, Bharwani KD, Stronks DL, Dirckx M, Huygen F (2022). Ketamine therapy for chronic pain in the Netherlands: a nationwide survey. Scand J Pain.

[6] Yang Y, Maher DP, Cohen SP (2020). Emerging concepts on the use of ketamine for chronic pain. Expert Rev Clin Pharmacol.

[7] Loix S, De Kock M, Henin P (2011). The anti-inflammatory effects of ketamine: state of the art. Acta Anaesthesiol Belg.

[8] Nan Z, Lihua Y, Peilin W, Zhongchun L (2021). Immunoregulation and antidepressant effect of ketamine. Transl Neurosci.

[9] Szalach LP, Lisowska KA, Slupski J, Wlodarczyk A, Gorska N, Szarmach J, Jakuszkowiak-Wojten K, Galuszko-Wegielnik M, Wiglusz MS, Wilkowska A (2019). The immunomodulatory effect of ketamine in depression. Psychiatr Danub.

[10] Zhao J, Wang Y, Wang D (2018). The effect of ketamine infusion in the treatment of complex regional pain syndrome: a systemic review and Meta-analysis. Curr Pain Headache Rep.

[11] Chitneni A, Patil A, Dalal S, Ghorayeb JH, Pham YN, Grigoropoulos G (2021). Use of Ketamine Infusions for Treatment of Complex Regional Pain Syndrome: A Systematic Review. Cureus..

[12] Perez R, Geertzen JHB, Dijkstra PU, Dirckx M, Van Eijs F, Frölke JP (2014). Updated guidelines complex regional pain syndrome type 1.

[13] Mangnus TJP, Dirckx M, Bharwani KD, de Vos CC, Frankema SPG, Stronks DL, Huygen F (2021). Effect of intravenous low-dose S-ketamine on pain in patients with complex regional pain syndrome: a retrospective cohort study. Pain Pract.

[14] Harden NR, Bruehl S, Perez R, Birklein F, Marinus J, Maihofner C, Lubenow T, Buvanendran A, Mackey S, Graciosa J (2010). Development of a severity score for CRPS. Pain.

[15] Goebel A, Birklein F, Brunner F, Clark JD, Gierthmuhlen J, Harden N, Huygen F, Knudsen L, McCabe C, Lewis J (2021). The Valencia consensus-based adaptation of the IASP complex regional pain syndrome diagnostic criteria. Pain.

[16] Cohen SP, Bhatia A, Buvanendran A, Schwenk ES, Wasan AD, Hurley RW, Viscusi ER, Narouze S, Davis FN, Ritchie EC (2018). Consensus guidelines on the use of intravenous ketamine infusions for chronic pain from the American Society of Regional Anesthesia and Pain Medicine, the American Academy of pain medicine, and the American Society of Anesthesiologists. Reg Anesth Pain Med.

[17] Chan AW, Tetzlaff JM, Altman DG, Laupacis A, Gøtzsche PC, Krleža-Jerić K, Hróbjartsson A, Mann H, Dickersin K, Berlin JA, Doré CJ, Parulekar WR, Summerskill WS, Groves T, Schulz KF, Sox HC, Rockhold FW, Rennie D, Moher D (2013). SPIRIT 2013 statement: defining standard protocol items for clinical trials. Ann Intern Med..

[18] Chan A-W, Tetzlaff JM, Gøtzsche PC, Altman DG, Mann H, Berlin JA, Dickersin K, Hróbjartsson A, Schulz KF, Parulekar WR (2013). SPIRIT 2013 explanation and elaboration: guidance for protocols of clinical trials. BMJ.

[19] Grieve S, Perez R, Birklein F, Brunner F, Bruehl S, Harden RN, Packham T, Gobeil F, Haigh R, Holly J (2017). Recommendations for a first Core outcome measurement set for complex regional PAin syndrome clinical sTudies (COMPACT). Pain.

[20] Hudak PL, Wright JG (2000). The characteristics of patient satisfaction measures. Spine (Phila Pa 1976).

[21] Herdman M, Gudex C, Lloyd A, Janssen M, Kind P, Parkin D, Bonsel G, Badia X (2011). Development and preliminary testing of the new five-level version of EQ-5D (EQ-5D-5L). Qual Life Res.

[22] Sigtermans MJ, van Hilten JJ, Bauer MCR, Arbous SM, Marinus J, Sarton EY, Dahan A (2009). Ketamine produces effective and long-term pain relief in patients with complex regional pain syndrome type 1. Pain.

[23] Noppers IM, Niesters M, Aarts L, Bauer MCR, Drewes AM, Dahan A, Sarton EY (2011). Drug-induced liver injury following a repeated course of ketamine treatment for chronic pain in CRPS type 1 patients: a report of 3 cases. Pain.

[24] Rolke R, Magerl W, Campbell KA, Schalber C, Caspari S, Birklein F, Treede RD (2006). Quantitative sensory testing: a comprehensive protocol for clinical trials. Eur J Pain.

[25] Kennedy DL, Kemp HI, Ridout D, Yarnitsky D, Rice ASC (2016). Reliability of conditioned pain modulation: a systematic review. Pain.

[26] Bharwani KD, Dirckx M, Stronks DL, Dik WA, Schreurs MWJ, Huygen F (2017). Elevated plasma levels of sIL-2R in complex regional pain syndrome: a pathogenic role for T-lymphocytes?. Mediat Inflamm.

[27] Bharwani KD, Dik WA, Dirckx M, Huygen F (2019). Highlighting the role of biomarkers of inflammation in the diagnosis and Management of Complex Regional Pain Syndrome. Mol Diagn Ther.

[28] Huygen FJPM, Niehof S, Klein J, Zijlstra FJ (2004). Computer-assisted skin videothermography is a highly sensitive quality tool in the diagnosis and monitoring of complex regional pain syndrome type I. Eur J Appl Physiol.

[29] Terwee CB, Roorda LD, de Vet HC, Dekker J, Westhovens R, van Leeuwen J, Cella D, Correia H, Arnold B, Perez B, Boers M (2014). Dutch-Flemish translation of 17 item banks from the patient-reported outcomes measurement information system (PROMIS). Qual Life Res..

[30] Dworkin RH, Turk DC, Revicki DA, Harding G, Coyne KS, Peirce-Sandner S, Bhagwat D, Everton D, Burke LB, Cowan P (2009). Development and initial validation of an expanded and revised version of the short-form McGill pain questionnaire (SF-MPQ-2). Pain.

[31] Sullivan MJL, Bishop SR, Pivik J (1995). The pain catastrophizing scale: development and validation. Psychol Assess.

[32] Nicholas MK (2007). The pain self-efficacy questionnaire: taking pain into account. Eur J Pain.

[33] Buscher HCJL, Wilder-Smith OHG, van Goor H (2006). Chronic pancreatitis patients show hyperalgesia of central origin: a pilot study. Eur J Pain.

[34] van Laarhoven AIM, Kraaimaat FW, Wilder-Smith OH, van de Kerkhof PCM, Evers AWM (2010). Heterotopic pruritic conditioning and itch – analogous to DNIC in pain?. PAIN®.

[35] Kriek N, de Vos CC, Groeneweg JG, Baart SJ, Huygen FJPM (2023). Allodynia, Hyperalgesia, (Quantitative) Sensory Testing and Conditioned Pain Modulation in Patients With Complex Regional Pain Syndrome Before and After Spinal Cord Stimulation Therapy. Neuromodulation..

[36] Yarnitsky D, Bouhassira D, Drewes AM, Fillingim RB, Granot M, Hansson P, Landau R, Marchand S, Matre D, Nilsen KB (2015). Recommendations on practice of conditioned pain modulation (CPM) testing. Eur J Pain.

[37] Olesen SS, Graversen C, Bouwense SAW, van Goor H, Wilder-Smith OHG, Drewes AM (2013). Quantitative sensory testing predicts pregabalin efficacy in painful chronic pancreatitis. PLoS One.

[38] Bosma RL, Cheng JC, Rogachov A, Kim JA, Hemington KS, Osborne NR, Venkat Raghavan L, Bhatia A, Davis KD (2018). Brain dynamics and temporal summation of pain predicts neuropathic pain relief from ketamine infusion. Anesthesiology.

[39] Niesters M, Aarts L, Sarton E, Dahan A (2013). Influence of ketamine and morphine on descending pain modulation in chronic pain patients: a randomized placebo-controlled cross-over proof-of-concept study. Br J Anaesth.

[40] Kirkpatrick AF, Saghafi A, Yang K, Qiu P, Alexander J, Bavry E, Schwartzman R (2020). Optimizing the treatment of CRPS with ketamine. Clin J Pain.

[41] Hakkaart-van Roijen L, Van der Linden N, Bouwmans C, Kanters T, Tan S (2016). Richtlijn voor het uitvoeren van economische evaluaties in de gezondheidszorg.

[42] Maher DP, Chen L, Mao J (2017). Intravenous ketamine infusions for neuropathic pain management: a promising therapy in need of optimization. Anesth Analg.

[43] Patil S, Anitescu M (2012). Efficacy of outpatient ketamine infusions in refractory chronic pain syndromes: a 5-year retrospective analysis. Pain Med.

[44] Schwartzman RJ, Alexander GM, Grothusen JR, Paylor T, Reichenberger E, Perreault M (2009). Outpatient intravenous ketamine for the treatment of complex regional pain syndrome: a double-blind placebo controlled study. Pain.

[45] Sheehy KA, Muller EA, Lippold C, Nouraie M, Finkel JC, Quezado ZMN (2015). Subanesthetic ketamine infusions for the treatment of children and adolescents with chronic pain: a longitudinal study. BMC Pediatr.

[46] Noppers I, Niesters M, Aarts L, Smith T, Sarton E, Dahan A (2010). Ketamine for the treatment of chronic non-cancer pain. Expert Opin Pharmacother.

[47] Xu J, Herndon C, Anderson S, Getson P, Foorsov V, Harbut RE, Moskovitz P, Harden RN (2019). Intravenous ketamine infusion for complex regional pain syndrome: survey, consensus, and a reference protocol. Pain Med.

[48] Sleigh J, Harvey M, Voss L, Denny B (2014). Ketamine–More mechanisms of action than just NMDA blockade. Trends Anaesth Crit Care.

[49] Kamp J, Van Velzen M, Olofsen E, Boon M, Dahan A, Niesters M (2019). Pharmacokinetic and pharmacodynamic considerations for NMDA-receptor antagonist ketamine in the treatment of chronic neuropathic pain: an update of the most recent literature. Expert Opin Drug Metab Toxicol.

[50] David Clark J, Tawfik VL, Tajerian M, Kingery WS (2018). Autoinflammatory and autoimmune contributions to complex regional pain syndrome. Mol Pain.

[51] Zhou Y, Wang C, Lan X, Li H, Chao Z, Ning Y (2021). Plasma inflammatory cytokines and treatment-resistant depression with comorbid pain: improvement by ketamine. J Neuroinflammation.

[52] Nikkheslat N (2021). Targeting inflammation in depression: ketamine as an anti-inflammatory antidepressant in psychiatric emergency. Brain Behav Immun Health.

[53] Birklein F, Ajit SK, Goebel A, Perez R, Sommer C (2018). Complex regional pain syndrome - phenotypic characteristics and potential biomarkers. Nat Rev Neurol.

