# Supplementary material for: Intermittent versus continuous esketamine infusions for long-term pain modulation in complex regional pain syndrome: protocol of a randomized controlled non-inferiority study (KetCRPS-2)
Source: BMC Musculoskelet Disord. 2023 Mar 29;24:239. doi: 10.1186/s12891-023-06258-4 (PMC10050795; doi:10.1186/s12891-023-06258-4)
Supplement: Supplementary file 1 — Additional file 1: Explanation of the full conditioned pain modulation protocol. [file 12891_2023_6258_MOESM1_ESM.pdf]

Supplementary file: Explanation of the full conditioned pain modulation protocol

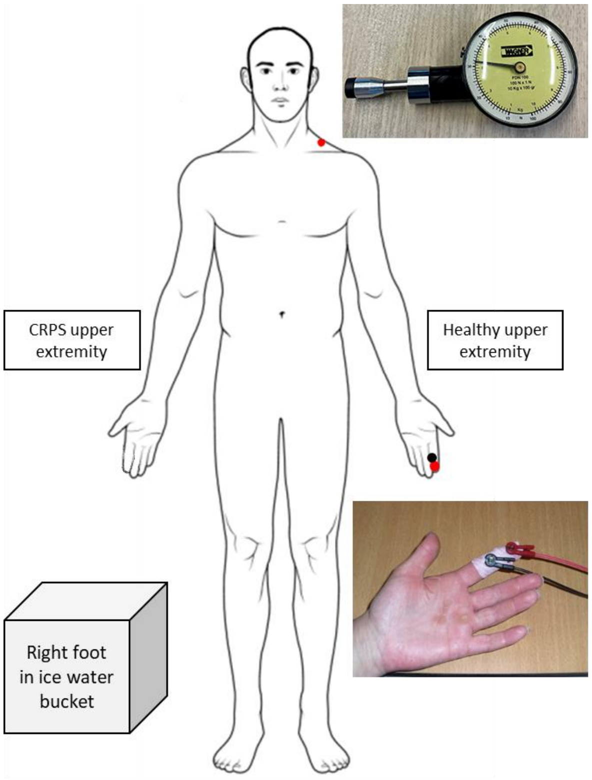

Figure 1. Conditioned pain modulation protocol consists of electrical stimulation and pressure algometry as test stimuli and ice water as conditioning stimulus. Figure adapted from Kriek et al. [3]

The conditioned pain modulation protocol used in this RCT is based on the protocol used at the pain department of Radboud University Nijmegen, The Netherlands [1, 2] and adapted from Kriek et al [3] at our department. All conditioned pain modulation tests are done in a standardized order. We use electrical stimulation and pressure algometry for the test stimuli. For electrical stimulation, the current perception threshold (CPT) and pain perception threshold (PPT) are determined three times with an ascending electrical stimulation protocol at the upper extremity at the contralateral side of the affected CRPS extremity. The conditioned pain modulation protocol is enriched with an additional test stimulus according to Yarnitsky et al. [4] by using pressure algometry at the trapezius muscle. For pressure algometry, the current perception threshold (CPT) and pain perception threshold (PPT) are determined three times with an ascending pressure algometry protocol. The ipsilateral healthy extremity of the CRPS affected extremity is submerged as the conditioning stimulus.

Patients receive instructions and training before conditioned pain modulation testing commences. Patients will be familiarised with the electrical stimulus and pressure used for conditioned pain modulation. With regard to electrical stimulation, all patients are in control of starting and stopping the electrical stimulation when a threshold is reached by pressing a handheld start/stop switch. The instructions given to the patients are as follows: 1) “start stimulation, but stop the stimulation as soon as you start to feel a tingling sensation at the test site” for the current perception threshold (CPT), 2) “start stimulation, but stop the stimulation when the stimulation becomes painful for the first time” for the pain perception threshold (PPT). Manual pressure algometry is used as a second test stimulus at the trapezius muscle. For the electrical stimulation and pressure algometry, an ascending intensity protocol

will be used and the CPT and the PPT will be individually calibrated. For the PPT, patients report a NRS pain score. This will be reported as PPT before ice water (PPT_{before}).

After a 20 minute brake, patients receive 3 consecutive electrical stimuli and stop the stimuli at the PPT and report the corresponding NRS pain score. This is followed by 3 pressure stimuli at the trapezius muscle. The manual pressure is stopped at the PPT and the corresponding NRS pain score is reported. After the 3 applied painful electrical and pressure stimuli, the ipsilateral healthy extremity is submerged in ice water (4 degrees Celsius) as the conditioning stimulus. The PPT value before ice water test (PPT_{before}) is compared with the PPT value after ice water test (PPT_{after}). The appropriate extremity is submerged into ice water and patients are instructed to remove that extremity if the pain from ice water becomes unbearable or when 2 minutes are passed. Three consecutive PPT_{after} measurements of electrical stimulation and pressure algometry are taken immediately following the removal of the extremity from the ice water. Furthermore, the duration of submergence in ice water is recorded.

Our analysis method for conditioned pain modulation is adapted from Olesen et al. [5] and Kriek et al. [3]. The PPTs will be assessed three times, and the median value of these three values will be calculated. By taking medians, outliers will not be taken in to analysis. Ratios will be used to explore the relationship of the threshold between the affected CRPS side and the healthy contralateral side [3, 5]. The advantage of using ratios is that they eliminate the intersubject differences in absolute CPT and PPT [3]. The ratios of each PPT threshold is calculated for all patients using the formula (threshold CRPS affected side) / (threshold healthy contralateral side) [3]. This yields the CPT ratio and PPT ratio of the electrical stimulation and pressure algometry.

Equipment electrical stimulation:

- Two self-adhesive disposable Ag/AgCl electrodes (Red Dot™, 3M Healthcare, St. Paul, MN, USA) are attached to the ventral side of the hand on the distal phalanx of the second digit on the contralateral healthy side. External electrical stimulation is applied to the skin via the Stmisol module (Biopac systems Inc., Goleta, California, USA) and is operated with a custom-built software program (not depicted in the figure). The constant current output consisted of block pulses delivered at 100 Hz, pulse width of 200 µs and an amplitude ramp of 1 mA/s. The current output is limited to 50 mA after which stimulation automatically stops. The current is delivered via a negatively charged electrode towards a positively charged electrode placed 2 cm apart.

Equipment pressure algometry:

- Wagner Force Dial FDK/FDN 100 SERIES Push Pull Force Gage. FDN 100/ 100N x 1N/ 10 Kg x 100gr.

References

1. Buscher HCJL, Wilder-Smith OHG, van Goor H: **Chronic pancreatitis patients show hyperalgesia of central origin: a pilot study**. *European journal of pain* 2006, **10**(4):363-370.
2. van Laarhoven AIM, Kraaimaat FW, Wilder-Smith OH, van de Kerkhof PCM, Evers AWM: **Heterotopic pruritic conditioning and itch – Analogous to DNIC in pain?** *PAIN®* 2010, **149**(2):332-337.
3. Kriek N, de Vos CC, Groeneweg JG, Baart SJ, Huygen FJPM: **Allodynia, Hyperalgesia, (Quantitative) Sensory Testing and Conditioned Pain Modulation in Patients With Complex Regional Pain Syndrome Before and After Spinal Cord Stimulation Therapy**. *Neuromodulation: Technology at the Neural Interface* 2022.
4. Yarnitsky D, Bouhassira D, Drewes AM, Fillingim RB, Granot M, Hansson P, Landau R, Marchand S, Matre D, Nilsen KB: **Recommendations on practice of conditioned pain modulation (CPM) testing**. *European journal of pain* 2015, **19**(6):805-806.
5. Olesen SS, Graversen C, Bouwense SAW, van Goor H, Wilder-Smith OHG, Drewes AM: **Quantitative sensory testing predicts pregabalin efficacy in painful chronic pancreatitis**. *PLoS One* 2013, **8**(3):e57963.
